# Over-the-Counter Drugs and Complementary Medications Use among Children in Southern Italy

**DOI:** 10.1155/2015/413912

**Published:** 2015-05-28

**Authors:** Claudia Pileggi, Valentina Mascaro, Aida Bianco, Maria Pavia

**Affiliations:** Department of Health Sciences, University of Catanzaro “Magna Græcia”, 88100 Catanzaro, Italy

## Abstract

The use of nonprescription medicines (NPDs) for children illnesses without a doctor's suggestion can lead to unnecessary medication use and is not free of risks. The aim of our study was to examine attitudes and practice of parents towards NPDs use for their children. We also investigated the conditions that may predict NPDs use. A cross-sectional survey was conducted on parents of children attending Community Based Pediatrician (CBP) consultation and data were collected through structured interviews. Positive attitude on NPDs use was reported by 71.4% of parents, and 61.5% of them had administered NPDs in the previous 6 months. Antipyretic drugs were the most frequently used medication class without the supervision of the CBP. A positive attitude towards NPDs was significantly more frequent in parents who did not use the CBP as the sole source of information about drugs. The study demonstrated a widespread use of NPDs in children in our context, supported by a substantial positive attitude towards their safety. However, considering potential harms related to some NPDs and the finding that most parents rely on CBP advice, role of CBP on appropriate use of NPDs by parents should be emphasized.

## 1. Introduction

Use of nonprescription drugs (NPDs), including over-the-counter drugs (OTCDs) and complementary alternative medicines (CAMs), is widespread all over the world as a first course of action for a range of childhood complaints [1, 2]. These include mild and moderate conditions, pain [3] and fever [4], and behavioral problems, such as irritability or sleeplessness [5, 6].

OTCDs are defined as safe and effective for use by the general public without a doctor's prescription [7, 8], whereas CAMs are a group of diverse medical and health care practices that are not considered part of conventional medicine [9]. CAMs are usually regarded safer than conventional medicines [10]. Parents may give CAMs to their child after having tried conventional medicines without success or in association with conventional medicines [11, 12]. The reason for CAMs use may also be the opportunity to have more options in the health care of children and to increase the likelihood that something would be helpful for the child [13, 14].

The use of NPDs is not free of risks, even if adverse effects of CAMs have been found to be minor and self-limiting [15, 16]. Inappropriate treatment of illness and symptoms can lead to unnecessary medication use and possible adverse effects [15, 17] if the parents' personal experience is not associated with the right information given by the physician [15].

In Italy, this should be not an issue since primary care is placed at the heart of the health care system. All residents are registered with a primary care physician (PCP) when they reach the age of 15 and, before this age, with a community based pediatrician (CBP). PCP and CBP provide various primary care services, such as health promotion and preventive activities, diagnosis, treatment, and follow-up of acute and chronic conditions. They also act as gate-keepers for access to secondary health services and for drug prescription for all patients in their list. These services are provided, within the National Health Service (NHS), to all patients free of charge, or at a minimal charge. When the PCP and CBP's office is closed Continuity of Care service provides night and weekend coverage as well as urgent home care to all patients. Finally, CBP can join a group practice with other CBPs so expanding access times to the study.

Since 2006 in Italy NPDs can be bought not only in pharmacies but also in supermarkets corners. This has raised some concern for the possibility of a more inappropriate use of these medications.

Parental attitudes toward children's medication have been studied quite widely [18], whereas there is a lack of literature investigating OTCDs and CAMs administration by parents in case of illness of their children and most studies have investigated management of specific diseases [5, 19–21] or have analyzed the use of a single category of NPDs: OTCDs [20, 21] or CAMs [16].

The primary aim was to examine attitudes and practice of parents towards NPDs use for their children. The secondary aim was to investigate the conditions, as sociodemographic characteristic, child health status, and source of information about drugs, which may predict NPDs use by parents.

## 2. Materials and Methods

### 2.1. Study Population

This cross-sectional study was conducted at waiting rooms of 8 CBPs in Catanzaro (Southern Italy), randomly selected from the list provided by the Local Health Units (LHU) including 37 CBPs that cover the healthcare needs of 56,520 pediatric patients.

From May 2013 through January 2014 all consecutive parents of ≤14 years old children attending CBP consultation and agreeing to participate were interviewed by trained physicians, not involved in patients care.

We investigated the use of both categories of NPDs: OTCDs and CAMs. Categories of drugs included in OTCDs were analgesics, laxative and antidiarrheal, cough and cold preparations, antihistamines, and dermatological, throat, and nasal preparations. As regard to CAMs we included several biologically based therapies as homeopathic and naturopathic medicines (e.g., calendula, royal jelly, and propolis), dietary supplements (including vitamin supplements), and probiotics. We excluded mind-body and manipulative therapies, such as meditation, chiropractic care, and yoga.

### 2.2. Review Instrument

The questionnaire included 27 questions divided into 4 sections. Each section elicited responses in a variety of formats: closed-ended questions with multiple answers possible, yes or no questions, and open option questions.

The first section explored sociodemographic characteristics of the parent. In the second section we investigated child health status. We used* SF-10 for Children* that is a 10-item instrument meant to be administered to the parents which measures eight domains of health and can be scored to produce physical and psychosocial health status summary scores [22]. This survey tool is intended for children between the ages of 5 and 18 years, and is available with a standard four-week recall period. Our population was also composed of parents of <5 years old children and, for such reason, we have chosen three questions of the SF-10 questionnaire (specifically questions numbers 1, 5, and 9) that could be asked to these parents too and could measure the perception of children's general health status. We also asked about chronic illnesses affecting the child and regularly used prescription drugs.

In the third part we investigated information about the use of OTCDs and CAMs without the CBP's suggestion in relation to child's ailments. Survey participants were first provided the following list of pediatric illnesses and symptoms: fever, upper respiratory infection/cold, sore throat, cough, vomiting, diarrhea, nausea, abdominal pain, dermatological problems (itchiness/redness of the skin, insect bites), allergies, wheezing, sleeping disorders, and headache. For each symptom parents were asked (1) whether their child had that illness or symptom in the previous six months, (2) whether they had administered an NPD, and (3) which NPD they had chosen.

Finally we explored sources of information concerning the child's medication and influences on medication use (e.g., CBP, internet, pharmacist, media, or personal experience). Informed consent was obtained from all responders, and the confidentiality of responses was assured. The questionnaire was pretested on a sample of parents to ensure clarity of interpretation and ease of completion to improve the validity of responses and the included information. Minor modifications on the sequence of questions and format were made on the basis of the pilot study. No medical records or interviews by any pediatrician were used as sources of data.

The study protocol was ratified by the Institutional Ethical Committee (“Mater Domini” Hospital of Catanzaro, Italy) (7 May 2013).

### 2.3. Statistical Analysis

Data were stored and analyzed using an appropriate database. Statistical analysis was performed using STATA software program, version 11 (Stata Corporation, College Station, Tx). Data were summarized using frequencies and percentages for categorical data and mean and standard deviations for continuous data. In the primary analysis we used the *t*-test (for continuous variables) and Pearson's chi-square (for categorical variables), to examine the association between NPDs use and several explanatory variables.

Multivariate logistic regression model was developed in order to describe the profile of parents who reported positive attitude on NPDs use for their children compared to those with a negative attitude (Model 1). Moreover, multinomial logistic regression analysis was used to investigate, in the subgroup of parents with positive attitude toward NPDs, determinants of the use of NPDs in presence of child's ailments. In particular, we have selected four target symptoms (cold, diarrhea, dermatological problems, and headache) among those reported by parents from the general list (Model 2). In this model the outcome variable was categorized into three levels: parents who did not utilize any drug when their child was ill, parents who utilized NPDs without the supervision of the CBP (baseline group), and parents who utilized NPDs only at the suggestion of the CBP.

Results are presented as odds ratio (ORs) and 95% CIs. All reported *p* values are two-tailed and a value <0.05 was considered statistically significant.

## 3. Results

The overall response rate was 98.6% resulting in 728 participating parents. Characteristics of the respondents and their children are fully described in Table 1.

The vast majority of respondents were mothers (89.3%) and the average age was 36.3 years (SD ± 6.9). Only 52.9% were employed. Mean age of children was 4.6 years (SD ± 3.4); 15.9% had a chronic illness diagnosed by a physician and 11.3% regularly used at least one prescribed medicine; antihistamines (74.4%) and corticosteroids (11%) were the most commonly prescribed drugs used, especially for allergic symptoms. More than half of the respondents preferred CBP as the only source of information about drugs.

Positive attitude on NPDs use without the supervision of the CBP was reported by 74.4% of the respondents, whereas 61.5% of them had administered NPDs to their children in the last 6 months. In this subgroup, antipyretic drugs were the most frequently used medication class without the supervision of the CBP. Paracetamol was the first choice drug for fever (95%) or headache (49%). More than half of the parents chose CAMs (especially herbal products) in presence of cough. Probiotic strains (e.g., lactobacilli spp.) were the most common products for gastrointestinal disorders (61%). For dermatological symptoms parents chose corticosteroid based drugs (22%) and CAMs (e.g., calendula,* Apis mellifica*) (14%). Vitamin supplements were utilized by 27% and herbal supplement (e.g. propolis, royal jelly) by 14% to strengthen children immune system. Results of the univariate analysis suggested that older parents (*χ* ^2^ = 20.66, 1 df, *p* < 0.0001), with lower education level (*χ* ^2^ = 5.44, 1 df, *p* = 0.02) and with higher number of children in the family (*χ* ^2^ = 87.6, 2 df, *p* < 0.0001), were significantly more likely to have a positive attitude towards NPDs use. Also, positive attitude was significantly higher in parents of older children (*t*-test = −5.75, 726 df, *p* < 0.001), with chronic diseases (*χ* ^2^ = 13.2, 1 df, *p* < 0.001), and that regularly used prescribed drugs (*χ* ^2^ = 12.9, 1 df, *p* < 0.001). Moreover, a positive attitude towards NPDs was significantly more frequent in parents who did not use the CBP as the sole source of information about drug characteristics (*χ* ^2^ for trend = 14.3, 1 df, *p* = 0.001), selection (*χ* ^2^ for trend = 29.11, 1 df, *p* < 0.001), and dose (*χ* ^2^ for trend = 16.72, 1 df, *p* < 0.001), who gather information about drugs before using them (*χ* ^2^ = 5.63, 1 df, *p* = 0.018), and who used dose recommendations provided on the product label (*χ* ^2^ = 19.7, 1 df, *p* < 0.001). Moreover, parents who used NPDs in the last 6 months were more likely to consult sources of information different from CBP (*χ* ^2^ = 48.06, 2 df, *p* < 0.001). When the multivariate logistic analysis was performed, a positive attitude towards NPDs use was significantly associated with higher number of children in the family (OR = 2.04, 95% CI = 1.50–2.78) and with the presence of limitations in the children activities because of physical health problems (OR = 3.28, 95% CI = 1.79–6.02); furthermore significant associations with information sources about medications were also confirmed (OR = 3.13, 95% CI = 1.62–6.04; OR = 2.9, 95% CI = 1.75–4.8) (Table 2).

303 parents were eligible to estimate NPDs use in presence of at least one of the target symptoms. Of them, 27% reported no drugs use, 58% NPDs without the supervision of the CBP, and 16% drugs only suggested by the CBP. Multinomial logistic regression model (Table 3) highlighted that choosing CBP as favorite source of information about drugs characteristics and always gathering information before administering the drug were significantly associated to no drugs use in presence of target symptoms, compared to NPD use without CBP supervision. Also, parents' perception of their children health status significantly influenced behaviors in presence of children illness. Indeed, parents who perceived limitations in children activities because of psychological health problems (RRR = 4.57, 95% CI = 1.66–12.58) and absence of limitations because of physical health problems (RRR = 0.26, 95% CI = 0.11–0.66) were more likely to use medications only after suggestion of the CBP.

## 4. Discussion

The results of this study showed that about two-thirds of parents had a positive attitude towards NPDs and have used them in the last six months for their children. 58.3% of parents used NPDs without CBP supervision in case of children's symptoms (cold, diarrhea, dermatological problems, and headache). Also, a vast majority of our sample showed a belief in self-care that might be favored by the opportunity to buy the drugs in the supermarket corner, where customers can choose medicines by themselves, as well as products like herbal remedies and cosmetics. These findings may appear in contrast with the organization of the Italian NHS in which all the children are assigned to a certain CBP since birth, without any charge, where PCP is the first figure consulted about health needs, because patients are mainly confident in professional skills and satisfied regarding the interpersonal relationship, and drug prescriptions are the most frequent outcome after a PCP consultation [23]. However, it may be hypothesized that the consequence of this favorable condition makes the parents confident in their ability to self-medicate their children. This is confirmed by the results of a recent survey that investigated parental and CBPs' knowledge and management of fever in Italian preschool children, which highlighted that there were not substantial differences regarding both correct and incorrect practices used by parents and by CBPs [24].

The prevalence of positive attitude of NPD in the present study was higher than that found among parents in other countries [10, 18]. However, these studies focused their interest on attitude about OTC medicines only, whereas we examined attitude of NPDs including both OTCDs and CAMs, since the difference between the two categories of medicines may be unclear to some consumers [25]. Moreover, the profile of parents with positive attitude towards NPDs was in accordance with that highlighted in previous studies [10, 18].

Our findings on NPDs use are in line with previous results highlighting that paracetamol was the most frequently used OTCD, in particular in children with fever or pain [19, 26, 27]. Even if paracetamol seems to be the drug of choice in the management of illnesses and injuries at home, it is also acknowledged to be the medication most frequently implicated in intentional and unintentional overdosing and liver toxicity [17, 28]. The process of safely administering medications is influenced by multiple factors, such as demographic characteristics of the parent [4, 21], number of other children [26], and parental perceived child health status [3]. Although it was not within the scope of this study to collect data relating to side effects of the drugs administered at home, the profile of the NPDs users in our survey is quite similar to that of previous studies and, therefore, it is reasonable to assume that the frequency of adverse events is comparable to that reported in the literature [17].

In our study use of CAMs was in agreement with a large survey conducted in the pediatric population in the United States [29], as regards to cough (53.9%) and dermatological manifestations (16.5%), whereas we found higher CAMs use among children with abdominal pain (25%).

Previous researchers have found that parents mostly rely on CBP as their main resource for medication information on how to manage children disease at home [15, 26], followed by package labels, pharmacists, and nurses [15, 19, 30]. However, as showed by multivariable analysis, parents with a positive attitude to NPDs use prefer other information sources than the CBP, such as internet or media, and so forth. The use of the Internet as a healthcare information resource has been described in the literature, showing that it is mostly used for information on general health, acute or minor illness, chronic conditions, and NPDs [31], although Eiland et al. found that parents do not greatly rely on it for information regarding their children health care [15]. The use of the Internet in this area is still controversial; it offers enormous opportunities, particularly for providing and improving consumer information with regard to health care. The major issue is that it represents an international and unregulated source of information, so parents should be able to interpret the medical ones. It is important to continue research on the use of the Internet among patients and their families. Moreover, institutions and physicians should better utilize web applications to improve patients education [32] and provide tools for them to distinguish the high-quality information. On the other hand, the availability of NPDs in nonpharmacy sources should stimulate CBPs to provide parents with in-depth information about drugs administration to their children and to ensure that parents have understood how to appropriately use OTCDs.

In our study, use of medications only after suggestion of the CBP was more frequent by parents having a worse perception of their children physical health. On the contrary, previous studies [33, 34] found that mothers thinking their children were susceptible to illness were more likely to regularly use NPDs.

Almost all of the questionnaires were filled in by mothers, which still represent the main educators in the family and the ones who take care of the family's health care [18]. Therefore, since it has been reported that women are more likely than men to seek self-treatment [35] and that subjects that use self-medication for themselves seem to be positively oriented also towards using it for their children [10, 36], women may represent the target population for educative programs on safe self-medication practices.

The results of our study should be interpreted in the light of few potential limitations. First, our survey was performed as cross-sectional and it is well known that cross-sectional design does not allow any cause-effect relationship and poses many problems in relation to hypothesis testing since data on “risk factors” and “outcomes” are assessed at the same time. Second, data were based entirely on patients self-reporting; however, we do not think that method of data collection may represent a problem because self-reporting is the only way to collect subjective information about various domains of perceived health status. Third, as is the case of all questionnaire surveys, another limitation is the potential recall bias. However, recall bias was mitigated by having restricted recall within a specified period.

We did not collect data relating to parents' medications use to identify whether it influenced self-medication for their children. However, as previously stated, the available studies [10, 36] showed positive attitudes to self-medication for their children in subjects practising self-medication for themselves. Moreover, we recruited parents through CBPs offices; perhaps parents who use NPDs for their children attend physician's offices less often. However, CBPs in Italy act as “gate keepers” for all children health demands in the NHS and therefore their consultation is widespread within the parents in Italy [30]. Finally, as in previous studies [3, 15], socioeconomic status was not analyzed in our survey, but there were only 15 parents (2.1%) that said medicines cost influenced their choice of products, which could mean that health problems are considered individual experiences more important than external factors and economic status. Anyway, recommending an appropriate medication that the parents can afford is imperative.

## 5. Conclusion

The study demonstrated a widespread use of NPDs in children in our context, supported by a substantial positive attitude towards their safety. However, considering potential harms related to some NPDs and the finding that most parents rely on CBP advice, role of CBP on appropriate use of NPDs by parents should be emphasized.

## Figures and Tables

**Table 1:** Distribution of the attitude of use of nonprescription drugs (NPDs) according to selected characteristics of the study population.

	Total (*N* = 728)	%	Positive attitude on NPDs^a^ use (*N* = 505)^b^	%
*Respondent *				
Mother	650	89.3	447	68.8
Other relatives (father, grandparents)	78	10.7	58	74.4
			*χ* ^2^ = 1.02, 1 df, *p* = 0.312	
Age (years)				
<35	304	41.8	183	60.2
≥35	424	58.2	322	75.9
			*χ* ^2^ = 20.66, 1 df, *p* < 0.001	
Marital status				
Married/cohabitees	707	97.1	488	69
Other (single, separated/divorced, or widow)	21	2.9	17	81
			*χ* ^2^ = 1.37, 1 df, *p* = 0.243	
Education level (years of schooling)				
<8	142	19.5	110	77.5
≥8	586	80.5	395	67.4
			*χ* ^2^ = 5.44, 1 df, *p* = 0.02	
Working activity				
No	343	47.1	241	70.3
Yes	385	52.9	264	69.6
			*χ* ^2^ = 0.24, 1 df, *p* = 0.621	
Number of children in the family				
1	330	45.3	171	51.8
2	312	42.9	263	84.3
≥3	86	11.8	71	82.3
			*χ* ^2^ = 87.6, 2 df, *p* < 0.001	
*Child *				
Gender				
Male	398	54.7	280	70.4
Female	330	45.3	225	68.2
			*χ* ^2^ = 0.4, 1 df, *p* = 0.527	
Age (years)				
0–2	219	30.1	116	53
3–6	341	46.8	256	75.1
7–11	133	18.3	107	80.5
>11	35	4.8	26	74.3
Mean ± SD	4.6 ± 3.4		5 ± 3.4	
			*t* = −5.75, 726 df, *p* < 0.001	
Chronic illness diagnosed by a physician				
No	612	84.1	408	66.7
Yes	116	15.9	97	83.6
			*χ* ^2^ = 13.2, 1 df, *p* < 0.001	
Prescribed medicine regularly used				
No	646	88.7	434	67.2
Yes	82	11.3	71	86.6
			*χ* ^2^ = 12.9, 1 df, *p* < 0.001	
General health perception by the parents				
Poor/fair	57	7.6	44	80.0
Good/very good/excellent	673	92.5	461	68.5
			*χ* ^2^ = 3.16, 1 df, *p* = 0.075	
*Source of information about medications *(*727; 99.9%*)^b^				
Favorite source of information about medications characteristics				
Only pediatrician	397	54.6	255	64.2
Pediatrician and other source^c^	294	40.4	218	74.2
Other source^c^	36	5	32	88.9
			*χ* ^2^ for trend = 14.3, 1 df, *p* = 0.001	
Influences on medications selection				
Only pediatrician	412	56.7	259	62.9
Pediatrician and other influences^d^	196	27	140	71.4
Other influences^d^	119	16.4	106	89.1
			*χ* ^2^ for trend = 29.11, 1 df, *p* < 0.001	
Use of product label or other kind of information before administering the drug				
No	172	23.7	132	76.7
Yes	555	76.3	373	67.2
			*χ* ^2^ = 5.63, 1 df, *p* = 0.018	
Use of dosing recommendations provided on the product label				
No	575	79.1	377	65.6
Yes	152	20.9	128	84.2
			*χ* ^2^ = 19.7, 1 df, *p* < 0.001	
Favorite source for dose information^e^				
Only pediatrician	492	85.6	307	62.4
Pediatrician and other source^c^	61	10.6	49	80.3
Other source^c^	22	3.8	21	95.5
			*χ* ^2^ for trend = 16.72, 1 df, *p* < 0.001	

^a^Nonprescription drugs.

^b^In brackets, the number and the percentage of the total sample of 728 subjects responding to the question.

^c^Internet, books, nurse, friends/family, pharmacist, media (newspaper/TV/magazines/radio), child care/teacher, or personal experience.

^d^Cost, packaging, advertising by media, pharmacist/physician/nurse endorsement, friend/family endorsement, child care worker, or personal choice.

^e^575 subjects were eligible, because they did not always use product label for dose information.

**Table 2:** Result of the logistic regression model for estimates of associations of attitude on nonprescription drugs (NPDs) with potential determinants of their use.

Attitude on NPDs^a^ use Log likelihood = −360.57, *χ* ^2^ = 125.28, 10 df, *p* < 0.001, and number of participants = 705				
Variable	OR	SE	95% CI	*p*

*Sociodemographic profile *				
Number of children in the family, continuous	2.04	0.32	1.50–2.78	<0.001
Age of respondent (<35 years old as reference)	1.20	0.23	0.82–1.75	0.35
Marital status (other^b^ as reference)	0.58	0.35	0.18–1.88	0.36
Education level (<8 years of schooling as reference)	0.81	0.21	0.49–1.33	0.40
*Children's health status *				
Limitations in schoolwork or activities with friends because of physical health problems (absence of limitations as reference)	3.28	1.01	1.79–6.02	<0.001
Prescribed medicine regularly used by the child (none as reference)	1.81	0.67	0.87–3.74	0.11
General health perception by the parents (poor/fair as reference)	0.69	0.28	0.30–1.55	0.36
*Source of information about medications *				
Influences on medications selection (only pediatrician as reference)				
Pediatrician and other source^c^	Backward elimination			
Only other source^c^	3.13	1.05	1.62–6.04	0.001
Utilization of product label or other information before administering the drug (no as reference)	0.73	0.17	0.47–1.16	0.18
Utilization of dosing recommendations provided on the product label (never/hardly ever/sometimes/often as reference)	2.90	0.75	1.75–4.80	<0.001

^a^Nonprescription drugs.

^b^Single, separated/divorced, or widow.

^c^Internet, books, nurse, friends/family, pharmacist, media (newspaper/TV/magazines/radio), child care/teacher, or personal experience.

**Table 3:** Result of the multinomial logistic regression model for estimates of nonprescription drugs (NPDs) use in the presence of at least one of 4 symptoms (cold, diarrhea, dermatological problems, and headache).

Outcome: NPDs^a^ utilization without supervision of the CBP^b^ Log likelihood = −264.20, *χ* ^2^ = 52.63, 34 df, *p* = 0.02, and number of participants = 303				
Variable	No medications users		Medications users at the suggestion of the CBP^b^	
	RRR (95% CI)	*p* value	RRR (95% CI)	*p* value

*Sociodemographic profile *				
Respondent (other^c^ as reference)	1.20 (0.37–3.86)	0.76	0.74 (0.17–3.19)	0.68
Age of respondent, continuous	0.96 (0.91–1.02)	0.18	1.00 (0.94–1.07)	0.89
Marital status (other^d^ as reference)	6.38 (0.71–57.4)	0.10	3.61 (0.39–33.31)	0.26
Education level (<8 years of schooling as reference)	0.77 (0.36–1.62)	0.49	0.92 (0.35–2.41)	0.86
Working activity (none as reference)	0.88 (0.48–1.63)	0.69	1.18 (0.56–2.49)	0.67
Number of children in the family, continuous	1.20 (0.78–1.84)	0.33	1.25 (0.75–2.11)	0.39
Child gender (male as reference)	1.09 (0.61–1.95)	0.76	0.64 (0.31–1.31)	0.22
Age of the child, continuous	0.98 (0.89–1.08)	0.66	0.95 (0.84–1.07)	0.38
*Children's health status *				
Parental general health perception (poor/fair as reference)				
Good/very good/excellent	2.67 (0.81–8.82)	0.11	1.89 (0.43–8.26)	0.40
Limitations in schoolwork or activities with friends because of physical health problems (absence of reference)	0.56 (0.27–1.15)	0.11	0.26 (0.11–0.66)	0.004
Limitations in schoolwork or activities with friends because of psychological health problems (absence as reference)	1.76 (0.65–4.78)	0.27	4.57 (1.66–12.58)	0.003
Child's chronic illness diagnosed by a physician (absence as reference)	0.78 (0.21–2.87)	0.71	0.52 (0.09–3.12)	0.48
Prescribed medicine regularly used by the child (none as reference)	1.37 (0.32–5.82)	0.67	3.06 (0.48–19.46)	0.24
*Source of information about medications *				
Favorite source of information about medications characteristics (only pediatrician as reference)				
Pediatrician and other source	0.46 (0.25–0.84)	0.01	1.05 (0.51–2.19)	0.89
Only other source^e^	0.25 (0.06–0.96)	0.04	0.63 (0.15–2.61)	0.52
Utilization of product label or other information before administering the drug (never/hardly ever/sometimes/often as reference)				
Always	2.23 (1.08–4.63)	0.03	1.24 (0.54–2.84)	0.61
Utilization of dosing recommendations provided on the product label (no as reference)	0.63 (0.29–1.35)	0.24	1.20 (0.51–2.81)	0.68

^a^Nonprescription drugs.

^b^Community based pediatrician.

^c^Father or grandparents.

^d^Single, separated/divorced, or widow.

^e^Cost, packaging, advertising by media, pharmacist endorsement, physician endorsement, nurse endorsement, friend/family endorsement, child care worker, or personal choice.
